# To smoothie or not to smoothie? A mixed-method approach of nutrition pilot intervention among individuals in opioid agonist treatment

**DOI:** 10.1186/s40795-025-01095-1

**Published:** 2025-07-04

**Authors:** Siv-Elin Leirvaag Carlsen, Einar Furulund, Elaheh Javadi Arjmand, Karl Trygve Druckrey Fiskaaen, Jørn Henrik Vold, Tesfaye Madebo, Torgeir Gilje Lid, Lars T. Fadnes, for the ATLAS4LAR and FruktBAR study group, Jørn Henrik Vold, Tesfaye Madebo, Mette Hegland Nordbotn, Maria Olsvold, Beate Haga Trettenes, Jan Tore Daltveit, Trude Fondenes, Per Gundersen, Marianne Cook Pierron, Christine Sundal, Maren Borsheim Bergsaker, Eivin Dahl, Tone Lise Eielsen, Torhild Fiskå, Marianne Larssen, Ewa Joanna Wilk, Mari Thoresen Soot, Rannveig E. Nesse

**Affiliations:** 1 Bergen Addiction Research, Department of Addiction Medicine, Haukeland University Hospital, Bergen, Norway; 2 Department of Global Public Health and Primary Care, University of Bergen, Bergen, Norway; 3 Centre for Alcohol and Drug Research, Stavanger University Hospital, Stavanger, Norway; 4 Division of Psychiatry, Haukeland University Hospital, Bergen, Norway; 5 Department of Clinical Science, University of Bergen, Bergen, Norway; 6 Department of Respiratory Medicine, Stavanger University Hospital, Stavanger, Norway; 7 Department of Public Health, University of Stavanger, Stavanger, Norway; 8 Oral health Centre of Expertise Rogaland, Stavanger, Norway

**Keywords:** Opioid agonist treatment, Nutrition, Mixed- method, Multicenter pilot study, Mental and somatic health

## Abstract

**Background:**

Patients with substance dependence typically have unhealthy diets and low degree of nutritional awareness. They frequently face nutritional deficiencies in both micro- and macronutrients, which can have a significant impact on their overall health. Incorporating more fruits and vegetables into their diet has been associated with reduction in diseases such as cardiovascular diseases and certain cancers. This mixed-method study aims to gain insight into participants’ experience and feasibility of a pilot intervention of receiving 250 ml/day of fruit smoothies for a six-week period.

**Methods:**

Individuals undergoing opioid agonist treatment in Bergen and Stavanger, Norway, were recruited for this multi-center pilot study. This study had a pre- and post-intervention design where an explanatory sequential design was employed, integrating qualitative methods to delve into patients’ experiences and perspectives. In addition, psychological distress (Hopkins Symptom Checklist (SCL-10), fatigue (three-item Fatigue Severity Scale (FSS-3)), and nutrition status (folic acid) were measured.

**Results:**

Twenty-four individuals with a mean age of 47 years (standard deviation: 8.6) participated. They expressed feeling more energetic and being more active, and some reported eating more meals than before and adding new food items to their diet. Oral health was a barrier to eating more vegetables and fruits while smoothies, on the other hand, were easy to consume even with poor dental status. There were no clear indications of changes in psychological distress (pre-test: 2.09 vs. post-test: 2.08) or fatigue (post-test: 4.19 vs. post-test: 4.43). Folic acid levels increased from 15.3 nmol/L at baseline to 17.0 nmol/L after the intervention.

**Conclusion:**

Receiving fruit smoothies seems to have several benefits for patients undergoing opioid agonist treatment, including helping them reflect on their health and diet and expressing more awareness about nutrition. Providing smoothies alongside patients’ opioid agonist treatment was seen as feasible and can be a door opener for clinicians to discuss nutritional issues in this population. To evaluate the effectiveness, a sufficiently powered randomized controlled trial is needed.

**Supplementary Information:**

The online version contains supplementary material available at 10.1186/s40795-025-01095-1.

## Background

Substance use disorder, and particularly opioid use disorders (OUD), is linked to several health risks, including nutritional deficiencies [1, 2]. Among people with substance use disorder, 61% preferred sugar and sugar-sweetened foods [3]. Individuals with opioid use disorders often exhibit significant nutritional inadequacies, such as unhealthy eating habits [4], weight loss, and even binge eating [5–7]. Besides, they often show a low interest in food, preferring quick and convenient food items that typically have low vitamin content [1, 4, 8]. They also consume fewer vegetables and fruits compared to the general population and often lack nutritional knowledge and food preparation skills [1]. Although opioid agonist treatment (OAT) is recognized to enhance the quality of life of patients with OUD [9, 10], infrequent meals, nutritional deficiencies, and convenience-oriented diet choices persist during treatment [8]. It appears that poor food intake patterns established during adolescence or the time of active substance use are retained, creating challenges when attempting to cease illegal opioid use. The majority of individuals with OUD have severe nutritional shortages of micro- and macronutrients, including vitamins such as vitamin B9 (folate) [11], vitamin D [12] as well as minerals like zinc, calium, magnesium, which impairs their ability to metabolize carbohydrates efficiently [1]. Moreover, higher consumption of fruits and vegetables is associated with a reduction in cardiovascular disease, cancers, and all-cause mortality [13].

Individuals with substance use disorder tend to have mental health comorbidities [14]. Studies have demonstrated the impact of nutritional interventions on mental health related symptoms [15]. A randomized controlled trial assessed the effect of a nutritional program among patients with major depressive episodes, and reported a substantial improvement in self-reported depressive and anxiety symptoms in the intervention group [16]. However, there is limited knowledge about nutritional interventions for individuals with opioid use disorder [17] and how such interventions impact them. To acquire deeper insight into this topic, the present study employs both qualitative and quantitative methodologies. We hypothesized that a structured dietary intervention would be perceived as meaningful and feasible by participants receiving OAT and that it could enhance awareness of diet and health. The primary objective is to explore participants’ experiences and assess the feasibility of the intervention. The study is not designed to evaluate their effects.

## Materials and methods

### Setting

A pilot study was initiated in Bergen and Stavanger, situated in Western Norway, with a combined population of 498 800 inhabitants [18]. Approximately 800 and 500 individuals, respectively, received OAT in 2022 in Bergen and Stavanger [19]. Individuals are eligible for OAT if they fulfil all six criteria for opioid dependence syndrome according to the International Classification of Diseases, version 10 [20], and they acquire their medication at the OAT outpatient clinics or having it delivered to their home by the OAT personnel, which occurs at least weekly. The OAT clinics are staffed with nurses, social workers, psychologists, and consultants in addiction medicine. The intervention took place at three outpatient OAT clinics in Bergen and one outpatient clinic in Stavanger. This pilot study is a part of the ATLAS4LAR project [21].

### Design

This was a six-week multi-center pilot study with a pre- and post-intervention design. Mixed method approaches help capture the intricacies of interventions in real clinical settings in health and care service assessments [22]. In the current study, we used an explanatory sequential design combining qualitative methods to understand patients’ experiences and perspectives with quantitative descriptions of physical test results [22, 23]. Quantitative data were collected first, followed by qualitative data collection.

A semi-structured interview guide was developed in collaboration with the authors, user representatives, and research nurses. Post-intervention interviews included questions about participants’ perceptions of the products, experiences during and after the intervention, and ideas for improvement. Descriptive statistics were used to provide basic information about the participants’ characteristics and test results from the 10-item version of the Hopkins Symptom Checklist (SCL-10) [24] (psychological distress), three-item Fatigue Severity Scale (FSS-3) [25], and folic acid levels as a biochemical indicator of nutritional status [11]. Folic acid and carotenoids are biochemical indicators of fruit and vegetable consumption, with studies demonstrating associations between these markers and increased longevity [26]. Additionally, prolonged deficient folate level has been associated with cognitive decline, neurological disorders, depression and certain types of cancer [27–29]. Insufficient intake of fruits and vegetables may constitute a risk factor for various diseases and premature mortality, whereas higher consumption is associated with multiple health benefits.

In Bergen, recruited OAT patients received a daily 250 ml bottle of fruit smoothie as a dietary supplement. The smoothies were selected based on availability, nutritional content, and assumed preference. They consisted of pureed fruits and berries, along with juice derived from these ingredients, with no added sugar and not made from concentrate. The smoothies provided 8–18% of the recommended daily allowance of folate, and did not contain milk, yogurt, or grains (see Supplementary Table 1 for details).

Participants were provided with seven pre-bottled smoothies per week in conjunction with OAT medication at outpatient clinics, with an oral agreement to consume one per day [17]. A variety of commercially available fruit smoothies were offered, featuring different combinations of apple, pineapple, mango, banana, orange, blueberry, passion fruit, coconut, lime, and blackcurrant. Participants could exclude specific options based on personal preference.

In Stavanger, participants attended smoothie workshops every other day, where they prepared freshly made smoothies while learning about different fruit and vegetable combinations. The workshop provided free access to a range of fruits and vegetables, including apples, beets, spinach, carrots, berries, and grapes, allowing all participants to experiment with different ingredient combinations. A healthcare professional and a member of the research team were present to offer assistance and answer questions. Participants consumed smoothies on-site and prepared a 250 ml bottle for the following day.

### Participants

Participants were recruited from four outpatient OAT clinic in Stavanger and Bergen. A total of 24 individuals, representing various age groups and both sexes, voulantarily enrolled in the smoothie pilot intervention. All participants had completed the intervention.

### Data collection and procedure

The interviews were conducted face-to-face in the period June-July 2021 at patient’s outpatient clinics. The study’s research nurses working at the outpatient clinics had direct contact with the participants and were responsible for recruitment and scheduled interviews. The researchers SELC, EF, and KTDF, who have experience with qualitative methodologies, performed the interviews. They had no relationships with the participants before the interviews. The interviews lasted 11 to 23 minutes and were transcribed verbatim. The participants were not compensated for their participation in the interview.

The Norwegian translation of the SCL-10 [24] was used to evaluate psychological distress among participants, while fatigue was assessed with the FSS-3 [25]. The research nurses collected venous blood samples from the participants at the baseline of the intervention, early May 2021, and at the end, end of July 2021, to analyze serum folic acid as measured as biomedical indicator of fruit intake, as well as collecting responses to the SCL-10 and FSS-3 questionnaires. The interviews and post-test data were obtained on the same day. Blood samples were collected according to standard protocol and sent to the Department of Medical Biochemistry and Pharmacology at Haukeland University Hospital in Bergen and the Department of Medical Biochemistry at Stavanger University Hospital in Stavanger (both accredited by ISO-standard 15189). The former laboratory assessed folate concentration in serum samples by means of the Electrochemiluminescence Immunoassay (10% analytical variation at concentration 8.4 nmol/L) [30], whereas the latter used the Chemiluminescence Microparticle Immunoassay (12% analytical variation at concentration 9 nmol/L) [31, 32].

### Data analysis

**Fig. 1:**
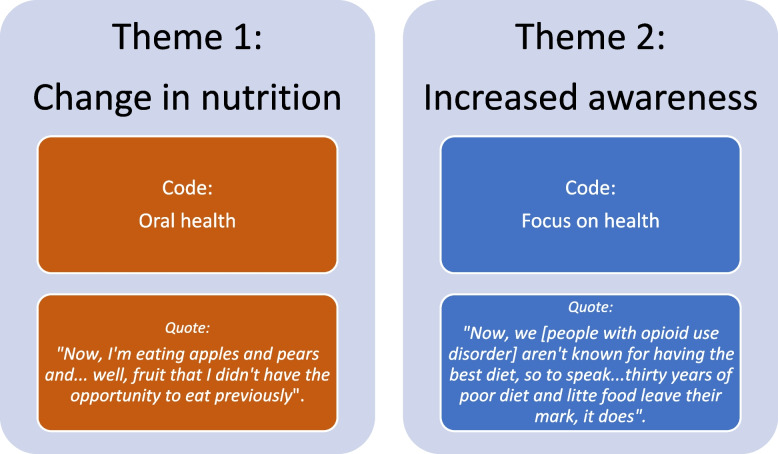
Qualitative analysis process

Following the guidelines set forth by Braun and Clarke [33], the researchers who collected the qualitative data carried out a data-driven analysis and an inductive thematic analysis to find, analyze, and report the major theme. Following transcribing, the first and second authors became acquainted with the transcripts through frequent readings. The first author then initially identified the dominant themes and created code descriptions. The second author revised the codes and themes, and the researcher discussed them, and a consensus was reached by eliminating or recoding equivalent themes. The analysis process is illustrated in Fig. 1. NVIVO [34] was used to generate the main categories and subcodes, and quotes used in this article were translated into English during the analysis process. To preserve anonymity, we have not included the participants’ real names but have used pseudonyms in the quotations.

Identified themes and example of codes and quoates are illustrtated in Fig. 1.

All the quantitative outcomes were analyzed using Stata-SE version 17 [35], employing descriptive statistics including proportions, mean scores, and standard deviations. As this is a pilot study not powered to evaluate effects but rather to indicate the degree of potential as well as experiences and feasibility, we do not test whether potential changes are significant.

All participants had signed a written informed consent form for the study. The study was approved by the regional ethical committee (no. 155386 REK south/east C, dated 23.09.2020/05.04.2022).

## Results

### Sociodemographic

This study included 24 participants, 10 women and 14 men, with a mean age of 47 years (range: 30 - 62 years). Thirteen individuals, nine men and four women, underwent interviews. All participants had a stable housing status in which they resided in their apartments, with slightly less than half living alone. None of the participants lived with their children. Regarding income, all participants received some form of social benefits, such as work assessment allowance or disability pension. The descriptive variables are shown in Table 1.

**Table 1:** Participants sociodemographic characteristics

**Characteristics**	**Frequencies, (IQR)** **Median, %** ***N*=24**		
Age, mean (SD)	47 (IQR: 42–53)		
Sex, female	10 (42)		
Education, completed			
Primary school (<10 years)	14 (58)		
Secondary or higher education (>10 years)	10 (42)		
Current living situation			
Stable housing^a^	24 (100)		
Living alone	11 (46)		
OAT medication			
Methadone	11 (46)		
Buprenorphine	13 (54)		
Lung disease			
Likely obstructive lung disease^b^	10 (42)		
Substance use last month	%	%	%
	Less than	1–3 days per week	4–7 days per week
Tobacco	*	*	91.7
Alcohol	70.8	29.2	-
Cannabis	41.7	16.6	41.7
Benzodiazepine	79.2	10.4*	10.4*
Stimulants	83.3	8.3*	8.3*
Opioids	91.7	*	*

Continuous variables are presented as median, while categorical variables are reported as count (n) and percentage (%). Percentages are calculated based on the total sample size (*n*=24). To preserve anonymity, responses with fewer than five participants are indicated with the symbol “*” instead of the exact percentage. IQR:P25-P75

^a^Stable housing typically refers to a dwelling, eighter an apartment or a house, that is owned or rented, where the individual has consistent, long-term living arrangements that provide safety and meet basic needs

^b^Likely obstructive lung disease: <0.70 for the forced expiratory volume in one second (FEV1)/forced vital capacity (FVC) measured by spirometry

Prior to the smoothie intervention, several participants reported having a poor diet, eating unhealthy foods such as sweets and fast food, or eating sparingly or rarely. When asked if the smoothie intervention had impacted their diet, four participants could not see signs of impact based on regular assessments from physicians, as John explained:

*“I’ve just been to the doctor and received blood pressure medicine and been told to start taking Vitamins, so it has not had any physical effect”.*

However, because of this intervention, most participants stated that they had experienced numerous changes. In the analysis, we identified the following two themes: changes in nutrition and increased awareness of the importance of fruits and vegetables in health.

### Changes in nutrition

Many participants ate only one meal a day; however, the smoothie intervention assisted some in improving their diet and meal routines. Some started incorporating them into their morning routine, finding that it provided an additional source of nutrients, while others noted that the intervention facilitated increased food consumption, particularly in terms of more frequent meals compared to their previous habits. In this context, Andrè conveyed that:

*“I now consume vegetables, meat, and a variety of other foods, rather than just ice cream, chicken, and coffee as I used to (...). Normally, I don’t have breakfast, but my stomach might start working, so I really want to eat. Maybe that’s why I started eating three eggs today”.*

Lillian added that:

*“It’s made me more conscious of things like eating a wider variety of foods and possibly eating a little more (food). It won’t be three times a week with fish, but I will eat some seafood on occasion”.*

Even though the tests did not show any substantial impact on health in terms of fatigue, or folic acid (Table 2), participants reported an enhanced appetite, increased energy, and more socialization with friends and family, as indicated by Henry’s comment:

*“I have a much better appetite and am doing a lot more physical activity. Previously, I was content with leisurely strolls followed by relaxation, but now I’m hiking and spending a lot more time with my landlord and family”.*

**Table 2:** Mean pre- and post-test scores with standard deviation (SD) and ∆scores (changes) for psychological distress (SCL-10), fatigue (FFS-3), and folic acid

Tests (n)	Pre-test Median (IQR)	Post test Median (IQR)	Diff change Median (IQR)
SCL-10 (*n*=16)	2.0 (1.65 – 2.35)	1.9 (1.35 – 2.85)	- 0.1 (−0.65 - 0.40)
FSS-3^a^ (*n*=14)	4.3 (2.00 – 6.67)	4.5 (2.00 - 7.00)	0.33 (−0.67 – 2.33)
Folic acid (*n*=16)	8.9 (7.55 – 21.95)	12.6 (7.95 – 26.10)	- 0.3 (−3.05 – 4.75)

^a^nmol/L. Clinical reference range of folic acid with <10 nmol/L considered likely deficient. Cut-off score for SCL-10: 1.85 (mean of all items), and cut-off for FSS-3: 4.0 (mean of all items). IQR:P25-P75

Some participants highlighted the association between diets and oral health, as well as the impact the smoothie had in this context. Some had dental difficulties that prevented them from eating fruits like apples. Several found the smoothies tasty and easy to consume, and as Celine noted, they were an excellent option for those with dental issues to receive additional vitamins from fruits.

*“I bought a smoothie machine, and now I drink fruit every day instead of chewing, which I can’t do because I don’t have teeth”.*

Furthermore, based on their favorable experiences with the smoothies, several participants purchased more smoothies in addition to those supplied in the intervention, while others acquired their own smoothie machines to create smoothies at home. Many participants intended to continue consuming smoothies following the intervention. However, fruit and vegetables are expensive in Norway, and a few participants mentioned that their finances were too tight to prioritize smoothies, as illustrated by Peter’s quote:

“*I am still poor. I started with multivitamins, but that was about it. But that is purely economic*”.

### Increased awareness

In terms of participant reflections on their health, many indicated that this intervention had made them more aware of their health. Consuming smoothies had made some participants more conscious of the importance of taking better care of themselves and their health, and several participants were particularly concerned about receiving enough vitamins, either through smoothies or by changing their diet. Marcus and Tommy stated that the intervention had changed their perspective on their own health:

* “I need to sharpen up a little since I’m fifty years old and it’s time. I’d love to enjoy some good years at the end of my life”*. Marcus

Tommy argued that drinking smoothies, in addition to taking folic acid, had a significant positive impact on his life, and he conveyed:

*“(...) and it’s now an upward spiral: the blood cells, vitamins, more and more food, and more activity. Because you see the favorable results, you want to do it [eat fruit and vegetables] more and more. It’s like a high”.*

Some, however, felt that the intervention should have been prolonged by a month or two. This extension, they argued, would facilitate the establishment of lasting changes in participants’ dietary perspectives, rather than being something they did only during this study period, as Cato stated:

*“.. don’t know if you get the full effect in a month, that is, a month is nothing (...) [It takes] another month or two to see if it truly is something or not”.*

## Discussion

This study found that, while there were no significant improvements in quantitative results, many participants reported positive changes. They experienced increased energy levels, which enabled them to be more social and active, and perceived the smoothies as a means of improving their overall nutritional intake and quality of life.

A recent systematic review identified inconsistent nutritional intake among individuals recieving OAT [2]. One factor influencing diet can be tobacco smoking. In our study, 91.7% of participants smoked tobacco and initially preferred fast food, as smokers tend to consume more high-fat foods and experience more frequent food cravings than non-smokers [36]. This may partly explain the low fruit consumption observed. Our findings align with a previous study in which 22 participants (92%) smoked daily, and smoking was considered an effective method of weight-control [7]. Similarly, a Norwegian study reported malnutrition rates ranging from 5% to 30% among people who injecting drugs [37], and many individuals with OUD often have a diet high in sweetened foods and characterised by sporadic eating [17, 38].

Although participants in our study were aware of the official dietary recommendations to consume at least five portions of fruits and vegetables daily, many found it challenging to do so. One reason may be limited nutritional knowledge, as many indivuduals who develop substance use disorders begin using substances during their adolescence and may not have learned what constitutes healthy foods or how to prepare them [7]. This lack of knowledge can persist, influencing their current food consumption, as they continue to maintain their unhealthy lifestyle behaviors [8]. Additionally, some perceived their diet as healthy and improved simply because they were now consuming more meals per day, in contrast to periods of active substance use when they often ate one or no meals.

A Portuguese study found that OAT patients failed to meet the recommendations for consumption of fruit, vegetables, and grains [39]. Furthermore, 40% rarely or never ate nuts, 28% never ate seafood, or rarely or never consumed vegetables, while they consumed more simple carbohydrates, contributing to a significant increase in calorie intake and potential weight gain [40]. In contrast, an Israeli study showed that OAT patients who received an intervention of two-hours of healthy diet education followed by six weeks of weight measurement significantly improved both nutrition knowledge and eating habits [6]. Thus, by providing smoothies as part of OAT, the staff at OAT clinics may be able to educate patients on the necessity of a balanced diet and enhance their understanding of what healthy eating entails.

Financial difficulties, faced by many OAT patients [41], may also be a major barrier to healthy eating and their ability to purchase fruits and vegetables. Therefore, fruit and vegetables are often deprioritized in favor of cheaper options. A Norwegian study supports this, reporting that 64% of individuals with active drug use cited a lack of money as a reason for reducing food intake [3]. In addition to financial barriers, poor oral health, highlighted by some participants in this study, futher complicated dietary choices. Individuals with OUD often experience diminished saliva production, which is required to prevent dental decay and gum infection, and individuals with long-term OUD may suffer from missing or decayed teeth. This can make chewing certain foods uncomfortable, leading them to favor softer foods [8]. Consequently, participants particularly appreciated the ability to consume fruits in liquid form, with many reporting an increase in fruit intake as a result of the intervention.

Beyond financial and oral health challenges, psychiatric disorders, such as depression, can also influence dietary habits. Depression is a prevalent among those who use opioids [42], and psychiatric and somatic comorbidities often co-occur [42–44]. A randomized controlled trial among adults with depression for the past two months or more, demonstrated that a Mediterranean diet, supplemented with fish oil, significantly improved mental health and led to increased consumption of vegetables, fruits, whole grain foods, nuts, and legumes [45]. These findings suggest that dietary interventions addressing both mental and physical health could improve nutrional outcomes for people recieving OAT.

Our intervention consisted of two approaches: one providing pre-bottled smoothies alongside OAT medication medication, while the other facilitated biweekley group workshops where participants prepared fresh smoothies together. Our findings suggest that distributing pre-made smoothies was the most practical approach, requiring fewer administrative and financial resources. In contrast, organizing smoothies workshops posed logistical challenges, particularly regarding participants attendance. This uncertanties were further compounded by the COVID-19 pandemic, with imposed restrictions on the number of individuals permitted to gather in the same space ([46] §13a). However, a key advantage of this approach was its social dimension. For some participants, gathering with other OAT patients to prepare smoothies served as an important social activity. Given the high prevalence of loneliness among OAT patients [47], these workshops provided a valuable social incentive. In contrast, the distribution of pre-made smoothies alongside OAT medication did not foster the same level of social interaction. However, this method ensured that all participants received their allocatied smoothies.

A challenge common to both approaches was verifying whether participants consumed the smoothies themselves or shared them with others. Some participants reported giving their smoothies to a partner or friend, which may have influenced their individual nutrition intake.

### Strengths and limitations

The COVID-19 pandemic was one of the pilot’s challenges. In Stavanger, the intervention included a workshop where participants made smoothies together. Due to the COVID-19 pandemic and subsequent restrictions, the smoothie workshops in Stavanger were limited to a maximum of 10 individuals, including study personnel. It is not unlikely that restrictions and fear of COVID-19 influenced attendance, as these workshops had few participants. Cooking lessons can be an excellent strategy to teach patients how to prepare and cook meals by covering topics such as cooking on a budget and boosting the intake of fruits and vegetables [48].

The self-recruitment of participants may introduce bias, as those who chose to participate may have had positive attitudes towards the intervention. However, interviews conducted by researchers not involved in patient care allowed for a more objective assessment of the intervention compared to scenarios involving a personal relationship between participants and interviewers.

Furthermore, we did not investigate if OAT-medication or the use of substances potentially altered their appetite. However, a qualitative study of ten OAT patients found that methadone was perceived as a suppressant to their appetite [8].

Although this study employed a mixed-methods approach, it was not intended for conducting an effectiveness evaluation. The pilot study aimed to assess the study’s degree of potential alongside experiences and feasibility, thus prioritizing the identification of trends over the assessment of effects.

A limitation of this study is that participants were not required to fast for eight hour prior to serum folate testing [49]. It has been suggested that a single hospital meal may normalize folate levels in individuals with true folate deficiency [50], raising the possibility that non-fasting could have provided false normal levels. However, a Norwegian study assessing folate status in OAT patients over a three years consistently found low folate levels that persisted throughout the study period [11]. This suggest that the impact of non-fasting on folate measurement may be less critical in this population, as their folate status appear to be chronically low, potentially overshadowing any transient or recent food intake. While the present study showed almost stable folate levels before and after the intervention, the absence of fasting could potentially have influenced the measurements. Our laboratory did not specify need of fasting before folate testing [51], although some laboratories recommend fasting, as serum folate concentrations can fluctuate significantly [49]. Notably, prolonged fasting may, counterintuitively, lead to an increase in plasma folate concentration rather than a reduction [52]. This suggests that while fasting status can influence folate measurements, its impact may not necessarily lead to lower values.

The study’s adaptability, shown throughout the COVID-19 pandemic, is one of its strengths. Clinicians switched from dispensing medication and smoothies at the outpatient clinic to delivering these to patients’ home. Despite the variability observed between patients’ physical test results and their subjective experiences of consuming smoothies, their perceptions could offer an ideal opportunity for lifestyle changes related to fruit intake. Altogether, this demonstrates that dispensing smoothies alongside medications poses few logistical challenges, even in a clinical routine that is perceived as hectic.

## Conclusions

This pilot study shows that the intervention of receiving smoothies for daily consumption was experienced as beneficial among people with OUD. Participants felt nourished, some expressed it as a better start to the day, and for some, drinking smoothies boosted their focus on what they ate and increased their nutritional awareness. People with dental health issues benefited greatly by being able to drink fruits as smoothies. There were indications of increased levels of folic acid post-intervention compared to pre-intervention. The pilot intervention also demonstrates the feasibility of giving smoothies to OAT patients in conjunction with medication delivery. Handing out smoothies was seen as a “door-opener” for the clinician to have a conversation with the patient about diet and nutritional risk factors. To evaluate the effect of such interventions, an adequately powered randomized controlled trial would be needed.

## Supplementary Information

Supplementary Material 1.

## Data Availability

No datasets were generated or analysed during the current study.

## Abbreviations

OAT: Opioid agonist treatment
OUD: Opioid use disorder(s)
SCL-10: Hopkins Symptom Checklist – 10
FFS-3: Three item Fatigue Symptom Scale
