# Supplementary material for: To smoothie or not to smoothie? A mixed-method approach of nutrition pilot intervention among individuals in opioid agonist treatment
Source: BMC Nutr. 2025 Jul 4;11:130. doi: 10.1186/s40795-025-01095-1 (PMC12228150; doi:10.1186/s40795-025-01095-1)
Supplement: Supplementary file 1 — Supplementary Material 1. [file 40795_2025_1095_MOESM1_ESM.docx]

Supplemented data – Nutrient content per 100 g.

| **Type of smoothies** | **Vitamin B9 (folate)** | **Energy (kJ)** | **Energy (kcal)** | **Fat** | **Carbohydrates** | **Protein** | **RDA*** | **Content per day (250 ml)** |
| --- | --- | --- | --- | --- | --- | --- | --- | --- |
| Pineapple & Mango | 15 µg | 195 kJ | 46 kcal | 0.2 g | 10.2 g | 0.4 g | 5% | 37.5 |
| Blueberry & apple | 9 µg | 211 kJ | 50 kcal | 0.2 g | 11g | 0.3 g | 2% | 22.5 |
| Raspberry & Strawberry | 23 µg | 189 kJ | 45 kcal | 0.2 g | 9.2 g | 0.5 g | 7% | 5 |
| Pineapple & coconut | 18 µg | 289 kJ | 69 kcal | 1.8 g | 12 g | 0.6 g | 5% | 45 |
| Mango & passion fruit | 17 µg | 225 kJ | 53 kcal | 0.2 g | 11.7 g | 0.5 | 5% |  |

Note: RDA: recommended Daily Allowance, * = recommended Daily Allowance in general population aged 18-70 years

Source: [www.matvaretabellen.no/en/search/?q=smoothies](http://www.matvaretabellen.no/en/search/?q=smoothies)
